# LCZ4r package R for local climate zones and urban heat islands

**DOI:** 10.1038/s41598-025-92000-0

**Published:** 2025-03-05

**Authors:** Max Anjos, Dayvid Medeiros, Francisco Castelhano, Fred Meier, Tiago Silva, Ezequiel Correia, António Lopes

**Affiliations:** 1 Department of Geography, Federal University of Rio Grande do Norte, Natal, Brazil; 2 Chair of Climatology, Institute of Ecology, Technische Universität Berlin, Rothenburgstraße 12, 12165 Berlin, Germany; 3 Institute of Geography and Spatial Planning (IGOT), Centre of Geographical Studies (CEG), University of Lisbon, Lisbon, Portugal; 4 Associate Laboratory Terra, Lisbon, Portugal

**Keywords:** Local climate zones, Urban heat islands, Software, LCZ4r, Crowdsourcing air temperature, Climate and Earth system modelling, Software

## Abstract

The LCZ4r is a novel toolkit designed to streamline Local Climate Zones (LCZ) classification and Urban Heat Island (UHI) analysis. Built on the open-source R statistical programming platform, the LCZ4r package aims to improve the usability of the LCZ framework for climate and environment researchers. The suite of LCZ4r functions is categorized into general and local functions (https://bymaxanjos.github.io/LCZ4r/index.html). General functions enable users to quickly extract LCZ maps for any landmass of the world at different scales, without requiring extensive GIS expertise. They also generate a series of urban canopy parameter maps, such as impervious fractions, albedo, and sky view factor, and calculate LCZ-related area fractions. Local functions require measurement data to perform advanced geostatistical analysis, including time series, thermal anomalies, air temperature interpolation, and UHI intensity. By integrating LCZ data with interpolation techniques, LCZ4r enhances air temperature modeling, capturing well-defined thermal patterns, such as vegetation-dominated areas, that traditional methods often overlook. The openly available and reproducible R-based scripts ensure consistent results and broad applicability, making LCZ4r a valuable tool for researchers studying the relationship between land use-cover and urban climates.

## Introduction

The Local Climate Zones (LCZ) classification provides a solid scientific framework for characterizing land surface areas, facilitating global studies on urban heat islands (UHI)^1^. Its utility is widely recognized, as evidenced by a growing body of research dedicated to investigating the relationship between 17-zone types and air temperature across different macroclimate regions^2–5^.

Existing methodologies and tools, such as the World Urban Database and Access Portal Tools (WUDAPT) initiative and the LCZ Generator web application, have successfully facilitated the mapping of urban landscapes into LCZs. The WUDAPT project offers standardized guidelines for collecting and analyzing urban morphology data worldwide, utilizing earth observation imagery (e.g., Landsat, Sentinel), training areas/polygons (TAs), Google Earth, and the System for Automated Geoscientific Analyses- SAGA software^6,7^. The LCZ Generator, a web-based tool, simplifies LCZ map creation by using TAs files and metadata, providing automated accuracy assessments and generating various LCZ-derivatives, such as total surface area^8^. While these tools have enhanced the global adoption of LCZs for scientific applications, they often require expertise in Geographic Information System (GIS) and can be time-intensive, posing a barrier for users without GIS knowledge.

While computational languages such as R^9^, Python^10^, and Matlab^11^ also require a basic understanding of command-line coding, they offer functionalities for data processing, statistical analysis, spatial analysis, and visualization, which simplify LCZ data analysis. These languages have emerged as accessible alternatives to the GIS-based system. However, the integration of the LCZ system and UHI modeling with these computational languages remains limited. For instance, the Python-based WUDAPT-to-WRF (W2W) package incorporates LCZ classes and their respective urban canopy parameters into the Weather Research and Forecasting (WRF) model^12^. On the R platform, the Lczexplore package compares LCZ maps from WUDAPT with GIS shapefiles from OpenStreetMap or the French BD Topo and provides the confidence level of LCZ quality classification^13^.

In this context, we introduce LCZ4r, a toolkit designed to streamline LCZ and UHI analysis within a computational language environment. LCZ4r enables users to work with LCZ data without requiring extensive GIS knowledge. Developed on the open-source R platform, renowned for its statistical computing capabilities^14,15^, LCZ4r leverages global and regional LCZ mapping efforts^16–18^ to provide a suite of functions for urban climate research. These functions allow users to extract LCZ maps for any landmass of the world at various scales, compute surface areas per LCZ class, and retrieve key physical parameters, such as fraction impervious surface, albedo, anthropogenic heat, sky view factor, terrain roughness, among others. Moreover, LCZ4r package generates time series of air temperature and anomalies between LCZ classes, performs spatial–temporal modeling of temperature readings, and calculates UHI intensity.

The LCZ4r tool brings other advantages by enabling users to interpolate data using LCZ as a background. The proposed LCZ-based interpolation incorporates LCZ system into the kriging model, considering not only the distances between measuring points but also the land use and cover patterns associated with LCZs. As a result, it can lead to better representativeness of spatial distribution of air temperature, particularly in urban areas with sparse measurements, compared to conventional interpolation methods that do not use LCZ data. Forthumore, LCZ4r automates the calculation of the UHI intensity using the LCZ framework, making it the first R package specifically designed for UHI modeling.

The development of the LCZ4r package aims to expand the application of the LCZ framework in climate research and urban planning. This paper presents an overview of the LCZ4r’s functionalities, showcasing its utility for UHI spatiotemporal analysis and encouraging researchers to incorporate this tool into their climate studies.

## Software design

The LCZ4r v0.1.0 is a suite of functions that encompasses various computational tasks, including data pre-processing^19–22^, geostatistical operations^23–25^, and visualization^26^. The LCZ4r is compatible with Windows, Ubuntu, and iOS operating systems, and it is recommended to use R integrated with RStudio^27^ for optimal performance. Users can access the latest features of the package by installing the development version from GitHub. To facilitate usage, LCZ4r provides documentation, user guidelines and tutorials at the website (https://bymaxanjos.github.io/LCZ4r/index.html).

**Fig. 1:**
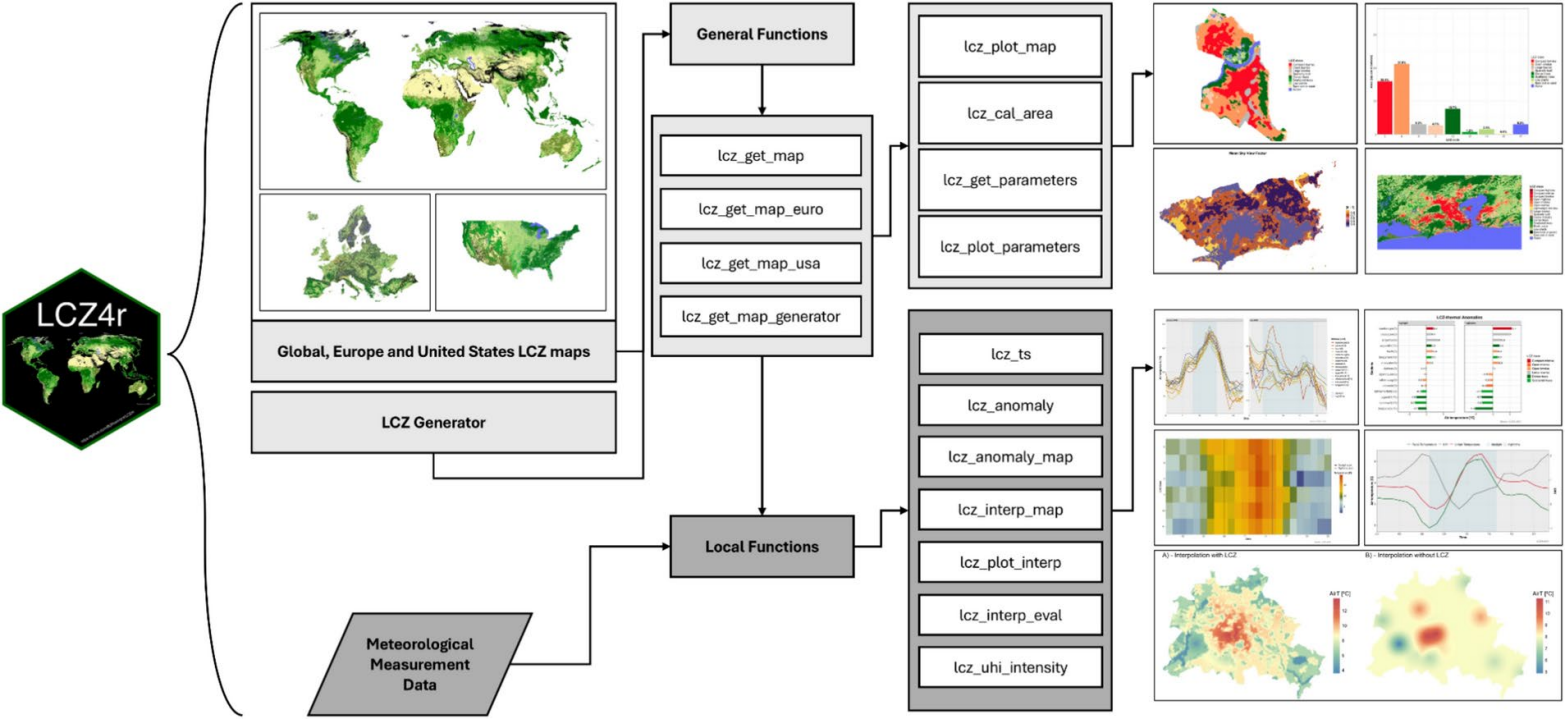
Structure of the LCZ4r package version 0.1.0.

The functions of the LCZ4r are assigned as lcz_*( ) and divided into two main groups: general and local functions (Fig. 1). General functions aim to facilitate the retrieval, analysis, and visualization of LCZ-related classes and urban canopy parameters, while local functions are designed to perform time series, anomalies, interpolation and UHI assessment using local datasets, such as air temperature readings from transect mobile and/or monitoring network. Table 1 summarizes all functions of LCZ4r.

**Table 1:** Summary of LCZ4r v 0.1.0 functions, their descriptions, inputs, and outputs.

Type	Function	Description	Input data	Output
General functions	lcz_get_map( )	Downloads an LCZ map for a specified city or region of interest	City name as a string or ROI in shapefile format	LCZ map raster (.tif)
	lcz_get_map_euro( )	Downloads an LCZ map for a specific area of interest within Europe	City name as a string or ROI in shapefile format	LCZ map raster (.tif)
	lcz_get_map_usa( )	Downloads an LCZ map for a specific area within the United States	City name as a string or ROI in shapefile format	LCZ map raster (.tif)
	lcz_get_map_generator( )	Retrieves LCZ maps from the LCZ generator platform	Identifier (ID)	LCZ map raster (.tif)
	lcz_plot_map( )	Creates a graphical representation of an LCZ map with standardized colors for each class	LCZ map raster (.tif)	Customized LCZ map (.png)
	lcz_cal_area( )	Calculates the area occupied by each LCZ class, expressed in both percentage and square kilometers	LCZ map raster (.tif)	Percentage areas (.png) and area data table (.csv)
	lcz_get_parameters( )	Extracts 12 Urban Canopy Parameters (UCPs) based on Stewart and Oke’s scheme (e.g., SVF, BSF)	LCZ map raster (.tif)	Parameters as raster stack (.tif) or shapefiles (.shp)
	lcz_plot_parameters( )	Generates a graphical map of UCP for visual analysis	UCP raster stack (.tif)	LCZ parameter map (.png)
Local functions	lcz_ts( )	Produces time series of air temperature data categorized by LCZ classes	LCZ map raster (.tif) and measurement data	Time series (.png) and data table (.csv)
	lcz_anomaly( )	Creates graphical representations of thermal anomalies based on LCZ classes	LCZ map raster (.tif) and measurement data	Anomaly plot (.png) and data table (.csv)
	lcz_anomaly_map( )	Generates spatial interpolations of thermal anomalies	LCZ map raster (.tif) and measurement data	Interpolated anomaly map (.tif)
	lcz_interp_map( )	Generates spatial interpolation map of air temperature data	LCZ map raster (.tif) and measurement data	Interpolated temperature map (.tif)
	lcz_interp_eval( )	Evaluates the accuracy of spatially interpolated LCZ maps	LCZ map raster (.tif) and measurement data	Evaluation results (.csv) and metadata as shapefile (.shp)
	lcz_plot_interp( )	Generates graphical representations of interpolated LCZ anomaly or air temperature data	Interpolated map raster (.tif)	Interpolated map plot (.png)
	lcz_uhi_intensity( )	Assesses Urban Heat Island (UHI) intensity based on LCZ classes	LCZ map raster (.tif) and measurement data	UHI intensity plot (.png) and data table (.csv)
Other functions	data(‘lcz_data’)	Provides hourly air temperature data for 17 meteorological stations in Berlin (2019–2020)	–	Temperature data table (.csv)
	lcz_get_map2( )	Integrates LCZ maps from external sources and clips it to a specific area	LCZ map raster (.tif) and city name as a string	LCZ map raster (.tif)
	lcz_get_tutorial( )	Opens web-based LCZ4r tutorials for general and local functions	Function type (general or local) as a string	LCZ4r tutorial webpage (.html)

### General functions

The lcz_get_map*( ) functions play a central role in the design of LCZ4r, as they facilitate the process of obtaining the LCZ map for a specified city or region of interest (ROI) without requiring TAs or GIS software, through simple line R command: my_map = lcz_get_map(city = “your city”). These functions access spatial feature data from OpenStreetMap (OSM) through the overpass API directly into R and Nominatim search engine to locate shapefiles https://nominatim.openstreetmap.org/ui/search.html (accessed on 24.03.2024). They then automatically import the global LCZ map version 3 from Zenodo repository (https://zenodo.org/records/8419340). The global LCZ map, developed by Demuzere et al.^18^, covers 410 regions of interest across 16 ecoregions worldwide. The global map is clipped to the intersected city or ROI, generating high-resolution (100 m) GeoTIFF raster with values representing the standard LCZ classes (1–17). Specific functions, such as lcz_get_map_euro( ) and lcz_get_map_usa( ), provide LCZ maps for Europe^28^ and Continental US^17^, respectively. The lcz_get_map_generator function extends this functionality by enabling access to cloud-optimized GeoTIFF LCZ maps hosted on the LCZ Generator Platform using a unique identifier (ID)^8^.

Although the lcz_get_map* functions offer a quick and convenient way to access LCZ maps, they rely on global and regional datasets, which can sometimes have reduced accuracy. In contrast, lcz_get_map_generator( ) function allows users to easily download a custom LCZ map that was previously created using the LCZ Generator. These custom maps are tailored to specific study areas and, when adequately implemented, can yield higher accuracy compared to large-scale products. This is particularly important for applications like air temperature interpolation, where map accuracy directly affects results.

Furthermore, these lcz_get_map*( ) functions serve as input for other functions in the LCZ4r. For example, the lcz_plot_map( ) utilizes the clipped raster to visually represent the LCZ map with colored standard classes. The lcz_cal_area( ) calculates the area occupied by each LCZ class, while the lcz_get_parameters( ) retrieves 12 Urban Canopy Parameters (UCP), including Sky View Factor (SVF), Building Surface Fraction (BSF), Pervious Surface Fraction (PSF), and Roughness Length class (z_0_), and converts these into shapefile or raster for spatial representation. These UCP maps are represented through maximum, minimum, mean values, e.g., the mean Sky View Factor (SVFmean) is derived from the range difference between maximum and minimum values. The parameters are based on reference ranges provided by Stewart and Oke^1^ for each LCZ class. The UCP available in the LCZ4r v. 0.1.0 can be accessed https://bymaxanjos.github.io/LCZ4r/articles/Introd_general_LCZ4r.html.

### Local functions

The LCZ4r package includes a suite of local functions designed for advanced analysis of air temperature data and related environmental variables. These functions require specific input data structures to operate efficiently. Typically, users must import air temperature data or other environmental variables into R as a structured data frame. This data frame should include columns for the date, meteorological station identifiers, and geographical coordinates of the station locations. It is important to ensure that the date-time format adheres to R conventions, such as “YYYY-mm-dd”, “2024-03-24 11:00:00”, or even “1/9/1986”. To assist users, the LCZ4r package provides a sample dataset, lcz_data(“lcz_data”), which contains air temperature measurements from Berlin and serves as a model for structuring data.

A key feature of the local functions is the flexibility they offer in temporal data selection, allowing users to tailor their analyses to specific timeframes. This is enabled by the cutData and selectByDate functions from the openair R package^20^. Users can aggregate data by year, season, month, day, or hour and filter specific periods, such as weekends, weekdays, or daylight hours. Seasons are determined based on the hemisphere of the LCZ map, while daylight periods are calculated using astronomical algorithms that estimate sunrise and sunset times^20^. This capability is particularly beneficial for analyzing diurnal temperature variations and UHI intensity.

The local functions facilitate detailed analyses of air temperature trends and variations across different LCZ classes. The lcz_ts( ) function generates time series of air temperature data categorized by LCZ classes, enabling researchers to monitor temporal trends. The lcz_anomaly( ) calculates temperature anomalies for each LCZ class. Here, an anomaly is defined as the difference between a LCZ station temperature and the mean temperature across all LCZ stations during a specific period. Thus, the anomaly at each LCZ station $T_{F(i)}^{\prime }$ is calculated using the following equation^29,30^:

1 $$T_{F(i)}^{\prime } = \underline {T}_{F(i)} - \frac{{\sum {\underline{ T}_{F(i)} } }}{n},$$

where $\underline {T}_{F(i)}$ is the mean (arithmetic) temperature of the urban LCZ stations and *n* is the number of LCZ stations. Positive temperature anomalies indicate that a particular LCZ is warmer compared to all other LCZs. This information can be important for assessing air temperature contrasts and identifying areas with deviations from the mean temperature within a study area.

Another key feature of the local functions is the geostatistical operations. Functions such as lcz_interp_map( ) and lcz_anomaly_map( ) are designed to map air temperature and anomalies values in an advanced way, as they operate under the assumption that temperature values obtained from sampled grid cells—where LCZ stations are deployed, can be extrapolated to non-sampled grid cells sharing the same LCZ class^29,30^. To do this, sample points are randomly generated on the LCZ map, ensuring each point receives an air temperature value and respective LCZ class. Subsequently, the sampled and non-sampled point values are interpolated using Kriging method. Universal Kriging incorporates the concept of randomness in spatial modeling of temperature variation. They are based on the assumption that temperature values measured at neighboring locations tend to be more alike than values measured at more remote sites. This method uses a variogram or covariance to quantify and interpret the spatial autocorrelation of data^24,25^.

Despite its widespread use for air temperature modeling, the Kriging method relies solely on the distances between the measurement sites to model temperature variation across the space. By incorporating LCZ information into the interpolation process, the lcz_anomaly_map( ) and lcz_interp_map( ) functions take into account factors such as land cover, urban form, and local changes induced by human activities, and this can result in a more representation of temperature anomalies and spatial temperature variations within urban areas. The lcz_interp_eval( ) function is designed to evaluate the interpolation outcomes at different spatial and temporal resolutions by comparing estimated maps with observed station data.

Regarding the UHI, the lcz_uhi_intensity( ) function provides two methods to calculate UHI intensity: LCZ and manual. In the LCZ method, the UHI is defined for a given time using the equation ^31^:

2 $$UHI = \underline{LCZ} x_{urban } - \underline{LCZ} y_{rural} ,$$

where $\underline{LCZ} x_{urban }$ and $\underline{LCZ} y_{rural }$ refer to the mean (arithmetic) air temperature values across all stations representing the urban and rural areas, respectively. This approach aligns with the recommendations proposed by Stewart et al.^31^, advocating for spatial averages to obtain locally representative values.

Identifying reference stations for urban and rural areas can be difficult in some cities, but LCZ classification itself can facilitate this task. For example, LCZ build types such as 1 (compact high-rise), 2 (compact mid-rise), 3 (compact low-rise), and 4 (open high-rise) typically signify densely populated areas with limited green spaces, making them ideal candidates for urban representation. Conversely, LCZ types B (scattered trees), C (bush, scrub), and D (low plants) or even LCZ 9 (sparsely built) and LCZ 6 (open low-rise) are more indicative of peripheral and rural areas. Specifically, the LCZ type A (dense tree) requires special attention, as it might complicate UHII analysis when studying micro air temperature variations caused by urban park effects.

In the LCZ method, the lcz_uhi_intensity(…, method = “LCZ”) automatically identifies the LCZ build types, starting from LCZ 1 and progressing to LCZ 8, to represent the LCZ*x*_*urban*_ area, whilst it starts from LCZ natural (B, C, D) to LCZ 9 (sparsely built) to represent the LCZ*y*_*rural*_ area. Once the LCZ types are determined, the function calculates the mean air temperature values for the selected $\underline{LCZ} x_{urban }$ urban and $\underline{LCZ} y_{rural }$.

While the automatic selection of LCZ types provides a standardized approach, we also recognize the importance of user judgment in UHII analysis. To accommodate this, the lcz_uhi_intensity(…, method = “manual”) offers a manual method, so that users have the freedom to select stations as references for the urban and rural areas. Using the manual method, the UHII is calculated for a given time based on the following equation:

3 $$UHII = T_{urban } - T_{rural} ,$$

where $T_{urban}$ and $T_{rural}$ represent the air temperature values for stations that represent the urban and rural areas, respectively.

## Results and discussion

### General functions for global LCZ access

**Fig. 2:**
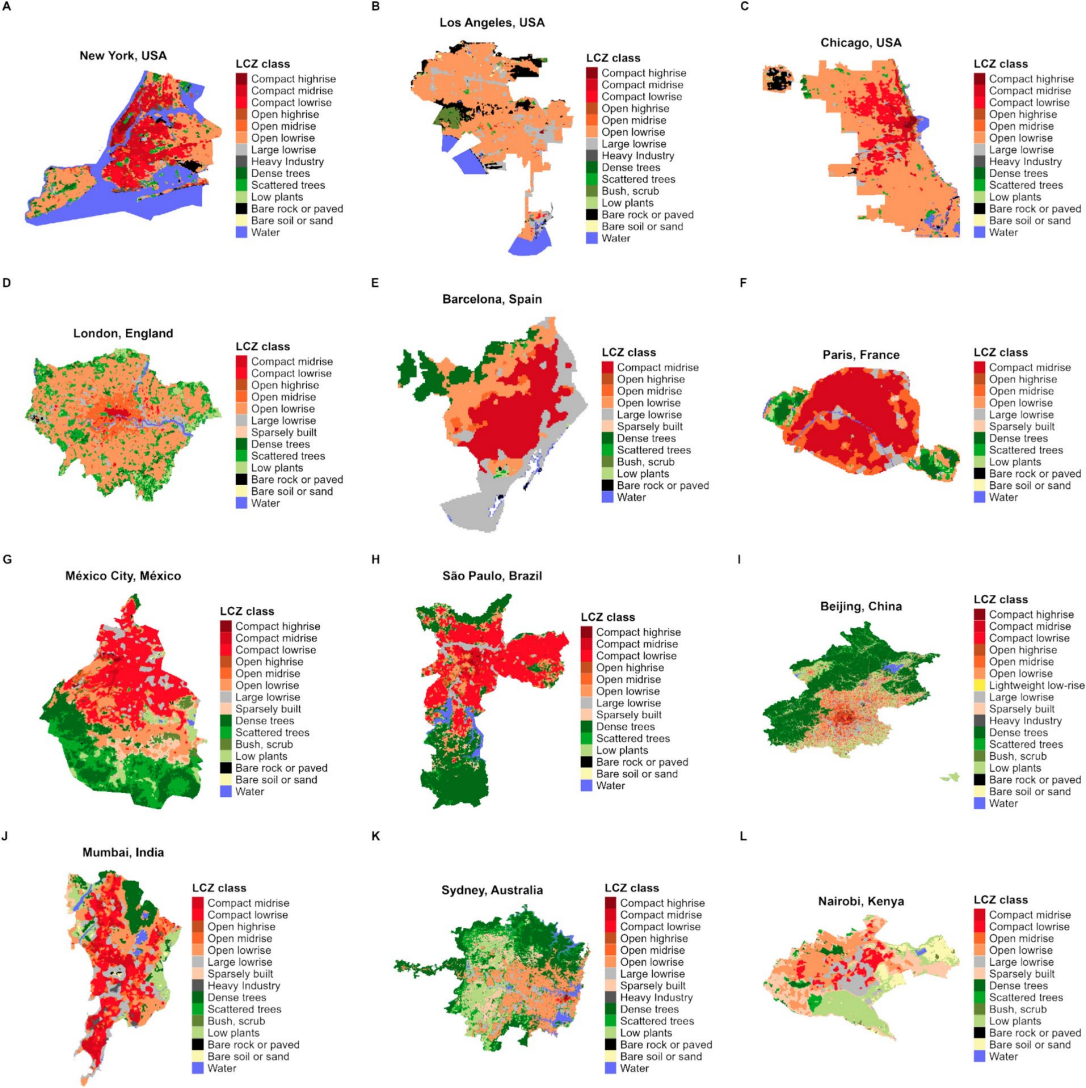
LCZ maps obtained with lcz_get_map( ), lcz_get_map_euro( ), and lcz_get_map_usa( ) functions from the LCZ4r package.

The primary purpose of the LCZ4r is to provide convenient and efficient access to LCZ maps. Figure 2 displays LCZ maps for cities in the continental US, Europe, Latin America, Asia, and Oceania, allowing a comparison between different LCZ classes across diverse land use-cover contexts.

**Fig. 3:**
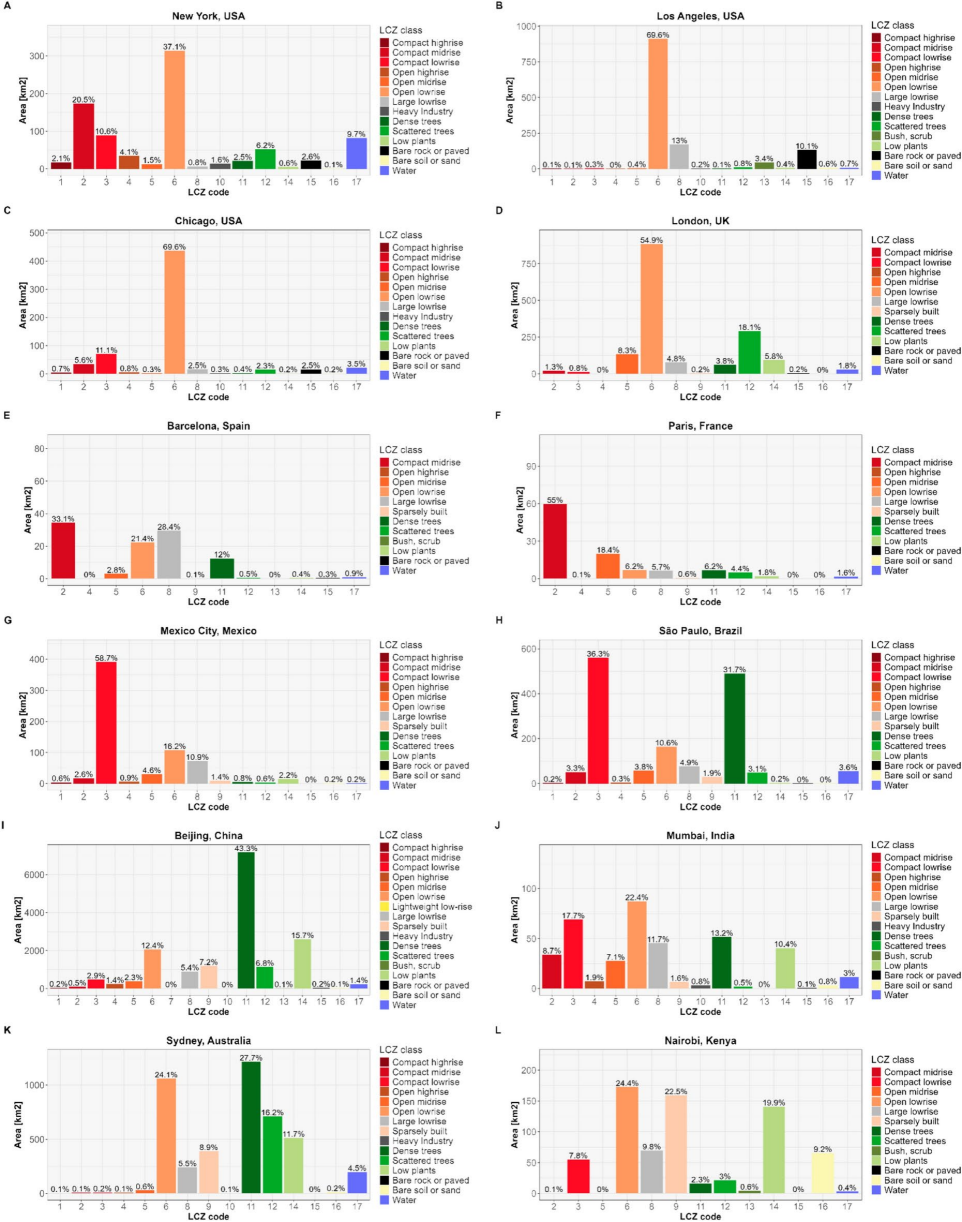
Percentage of area covered by each LCZ class in square kilometers calculated with lcz_cal_area( ) function from LCZ4r package.

Figure 3 presents the calculated percentage areas in square kilometers for each LCZ class. It enables the users to assess the spatial extent of different LCZ typologies and landscape composition, facilitating comparison across diverse urban and non-urban environments. For instance, LCZ 6 (Open low-rise) exhibits larger coverage in metropolitan areas such as New York, Los Angeles, Chicago, and London, while LCZ 2 (Compact midrise) predominates in cities like Barcelona and Paris, and LCZ 3 (Compact low-rise) in cities such as Mexico City and São Paulo. Qi et al.^32^ analyzed LCZ composition ratios across six US metropolitan areas from 1986 to 2020 and found a consistent decline in natural class proportions, describing just 11% by 2020 due to urban expansion. During this period, LCZ 6 presented the most significant growth, increasing by 20%. This growth primarily came to the expense of LCZ 9 (sparsely built), LCZ C (bush, scrub), LCZ D (low plants), and LCZ F (bare soil or sand), which contributed to 8%, 7%, 3%, and 2%, respectively.

**Fig. 4:**
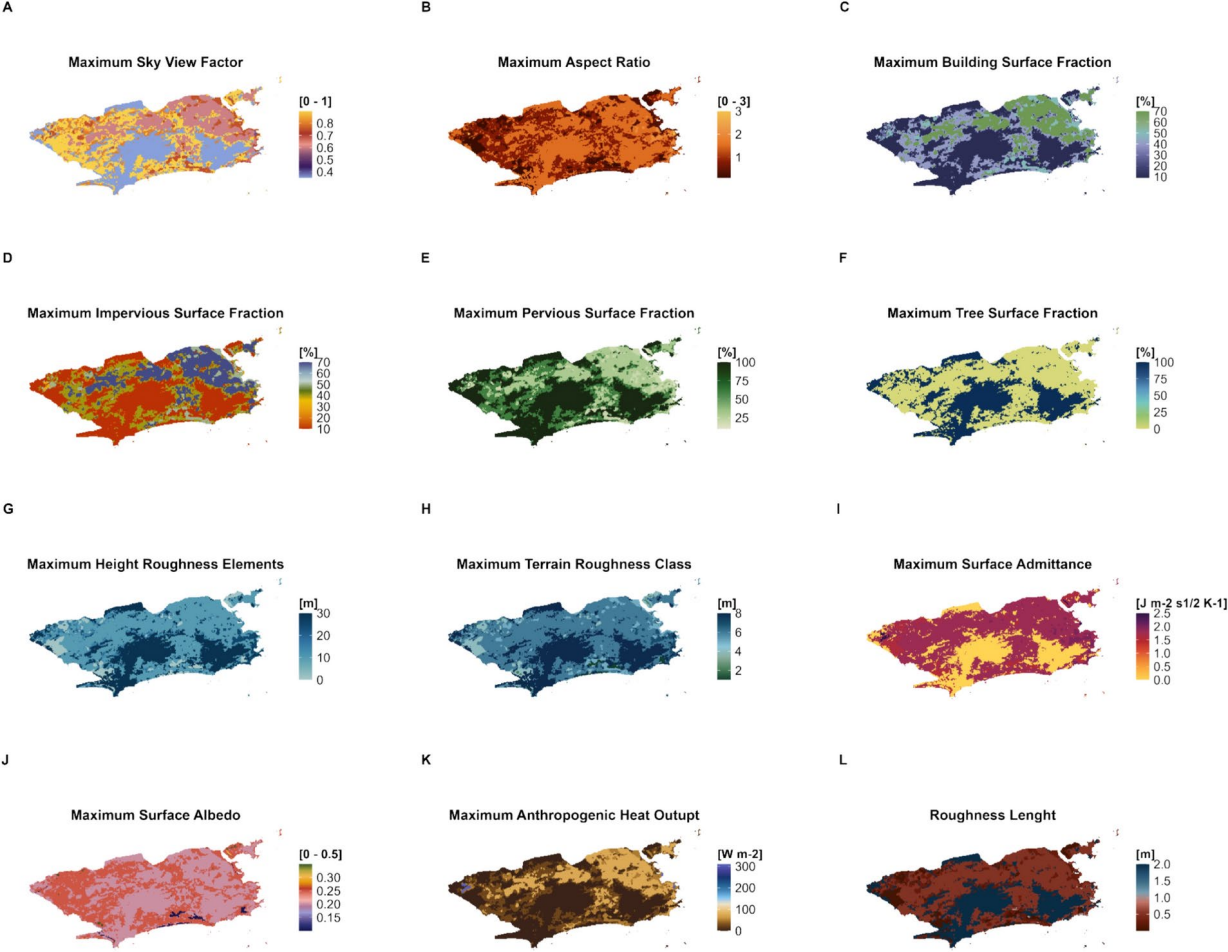
Maximum LCZ-related physical parameter maps aggregated 100 × 100 m for Rio de Janeiro city. They were generated by using the lcz_plot_parameters( ) function from the LCZ4r package.

Figure 4 presents a series of UCP maps for Rio de Janeiro City. These maps depict surface structure, surface cover, surface material and human activity attributes. LCZ4r transforms the reference range values proposed by Stewart and Oke^1^ for various physical properties into maps with a 100-m horizontal resolution. The LCZ-related parameter maps can serve as inputs for many modeling applications and statistical climate analysis. For example, the pervious surface fraction is often used in empirical-biogenic models to estimate fluxes of CO_2_ in urban and forest environments^33^; the SVF is widely applied in UHI assessments^34^; the surface albedo, on the other hand, is often related to air pollutant concentrations^35^.

### Local functions for air temperature studies

To demonstrate the capabilities of the local functions for analyzing intra-urban air temperature and UHI intensity, we selected four cities: Berlin, Lisbon, Curitiba, and Aracaju (Table 2). These cities were chosen based on the availability of data from urban meteorological networks and campaign monitoring efforts. It is important to note that LCZ4r does not directly assess the quality of LCZ maps or datasets. Instead, it assumes that accurate LCZ mapping has been previously assessed^16–18^ and that the datasets used in Berlin^36,37^, Lisbon^38,39^, Curitiba^40,41^ and Aracaju^42,43^ are appropriate for UHI analysis.

**Table 2:** Datasets used for application of local functions in this study.

City, country	Climate type	Kope classification^44^	Data	Period	Number of stations
Berlin, Germany	Temperate	Dfb	Hourly mean air temperature (°C)	1 Jan 2019–31 Dec 2020; 1 Jan–Dec 2018	23 Meteorological stations; 2474 Citizen weather stations (CWS)
Lisbon, Portugal	Mediterranean	Csa	Hourly mean air temperature (°C)	1 Jul 2021–31 Aug 2021	15 Meteorological stations
Curitiba, Brazil	Subtropical	Cfb	Hourly mean air temperature (°C)	1 Oct 2015–15 Aug 2018	08 Meteorological stations
Aracaju, Brazil	Tropical	Am	Hourly mean air temperature (°C)	1 Oct 2014–30 May 2016	06 Meteorological stations

#### Time series

**Fig. 5:**
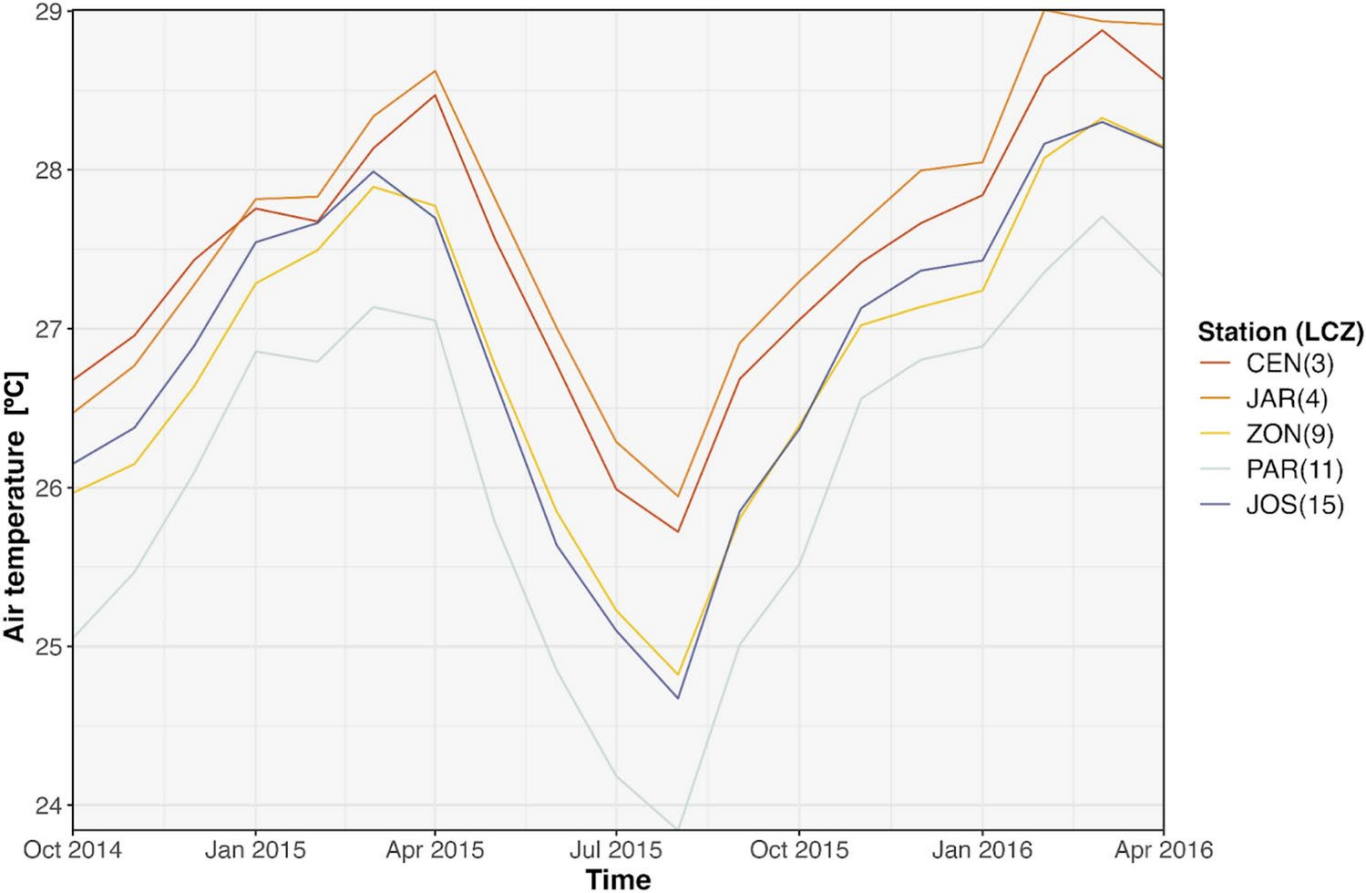
Time series of monthly air temperatures for various LCZ classes in Aracaju (2014–2016), generated using the lcz_get_map( ) and lcz_ts( ) functions from the LCZ4r package. The legend identifies the monitoring stations and their corresponding LCZ class codes.

A practical use of the LCZ4r using air temperature data from Aracaju is presented in Fig. 5. It integrates LCZ information for each meteorological station, allowing the characterization of the temperature regimes of different LCZ classes over time. The outcome clearly shows distinct thermal patterns associated with LCZ, mainly the CEN and JAR stations, classified as LCZ 3 (Compact Low-Rise) and LCZ 4 (Open High-Rise), respectively. These stations, located in central areas,consistently exhibit higher air temperatures compared to other LCZ classes, such as PAR (LCZ 11—Dense tree), situated in an urban park.

**Fig. 6:**
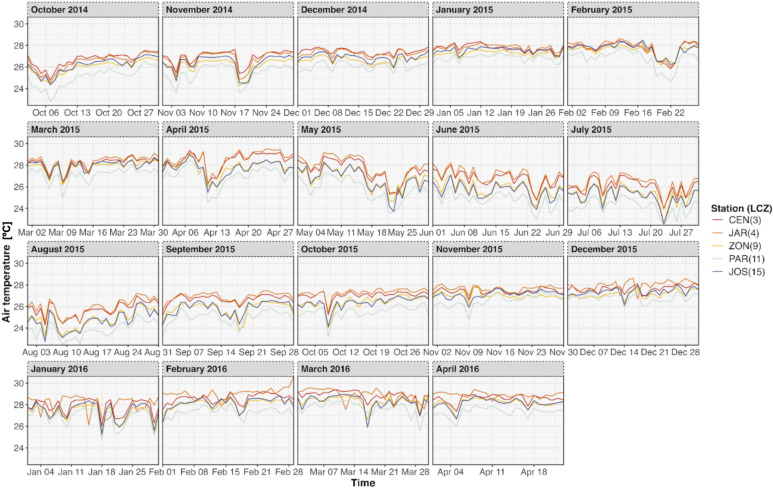
Time series of daily air temperature by month and year for different LCZ classes in Aracaju (2014–2016), generated by the lcz_get_map( ) and lcz_ts( ) functions from the LCZ4r package. The legend identifies the monitoring stations and their corresponding LCZ class codes.

The LCZ4r provides flexible temporal analysis, allowing users to explore air temperature variations across different time scales. Options include daytime and nighttime comparisons, seasonal trends, and even hourly fluctuations within a single day by activating the temporal selection argument by. For instance, Fig. 6 illustrates daily air temperature between LCZ classes, separated by month, over the 2014–2016 period.

#### Anomaly

**Fig. 7:**
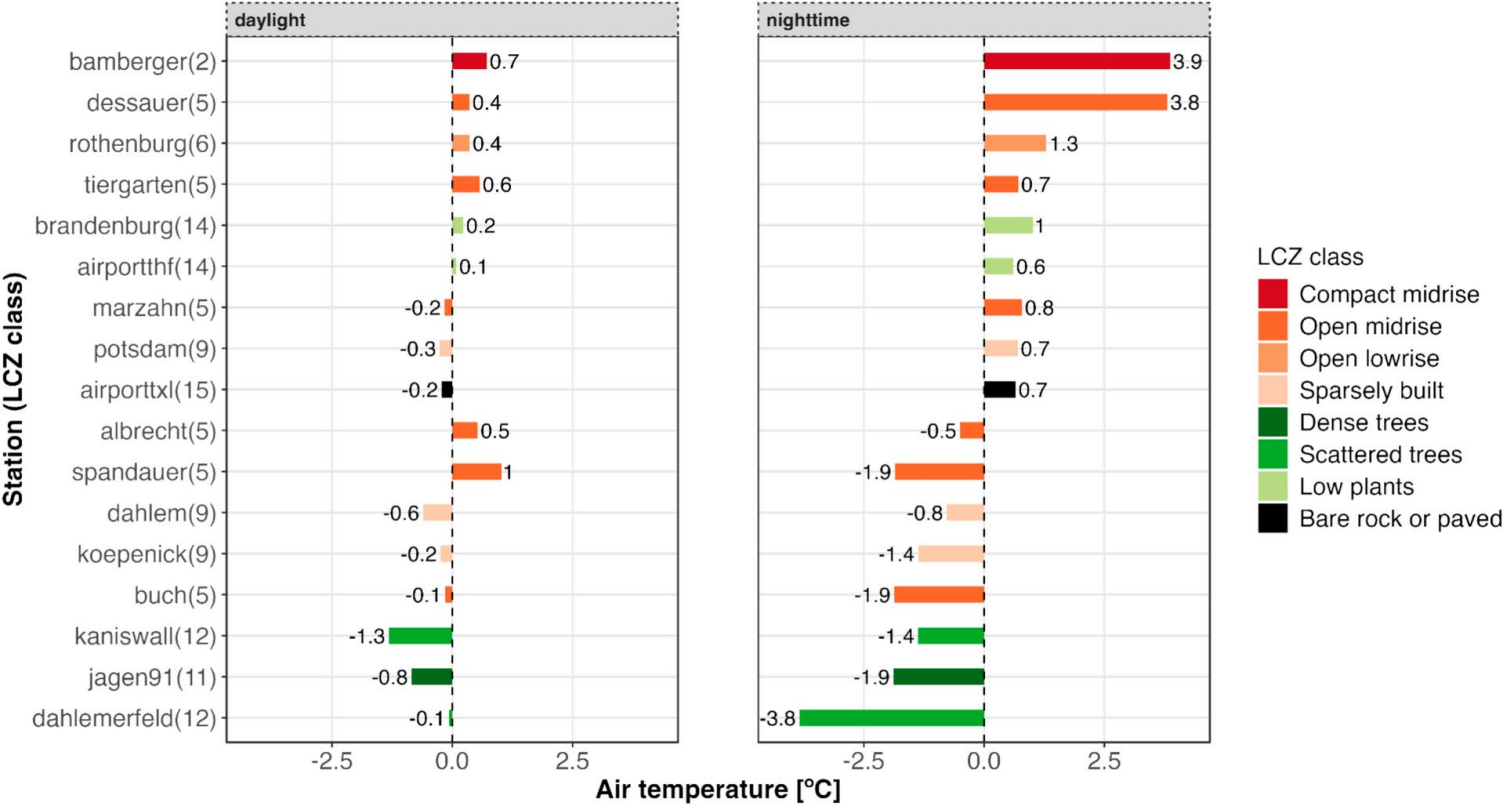
Hourly air temperature anomalies across different LCZ classes, divided into daylight and nighttime periods, in Berlin on May 29, 2019. The plot was generated using the lcz_get_map_generator( ) and lcz_anomaly( ) functions from the LCZ4r package. The legend identifies the meteorological stations and their respective LCZ class codes.

Figure 7 presents the air temperature anomalies for Berlin on a specific spring day, differentiated between daytime and nighttime periods. These anomalies are based on deviations of observed temperatures from a specific-term average at each station in the dataset. The LCZ4r normalizes temperature variations, accounting for inherent differences in baseline temperatures associated with distinct LCZ classes.

The anomalies show contrasts between LCZ classes, highlighting the influence of land use and cover on urban temperature patterns. For example, during the nighttime period, stations classified as LCZ 2 (Compact midrise) and LCZ 5 (Open midrise) exhibited positive anomalies (+ 3.9 °C and + 3.8 °C, respectively), indicating higher nocturnal temperatures in densely built urban areas. In contrast, LCZ 11 (Scattered trees) and LCZ 12 (Dense trees) stations recorded negative anomalies (− 1.9 °C and − 3.8 °C, respectively), reflecting cooler nighttime temperatures in areas with abundant vegetation. Daytime anomalies, while less pronounced, still demonstrate differences. Stations in built-up areas (e.g., LCZ 2 and LCZ 5) exhibited slight positive anomalies (+ 0.7 °C to + 1.0 °C), whereas stations in vegetated areas like LCZ 11 (− 0.8 °C) and LCZ 12 (− 1.3 °C) had negative anomalies, emphasizing their cooling effects.

#### Modeling UHI

**Fig. 8:**
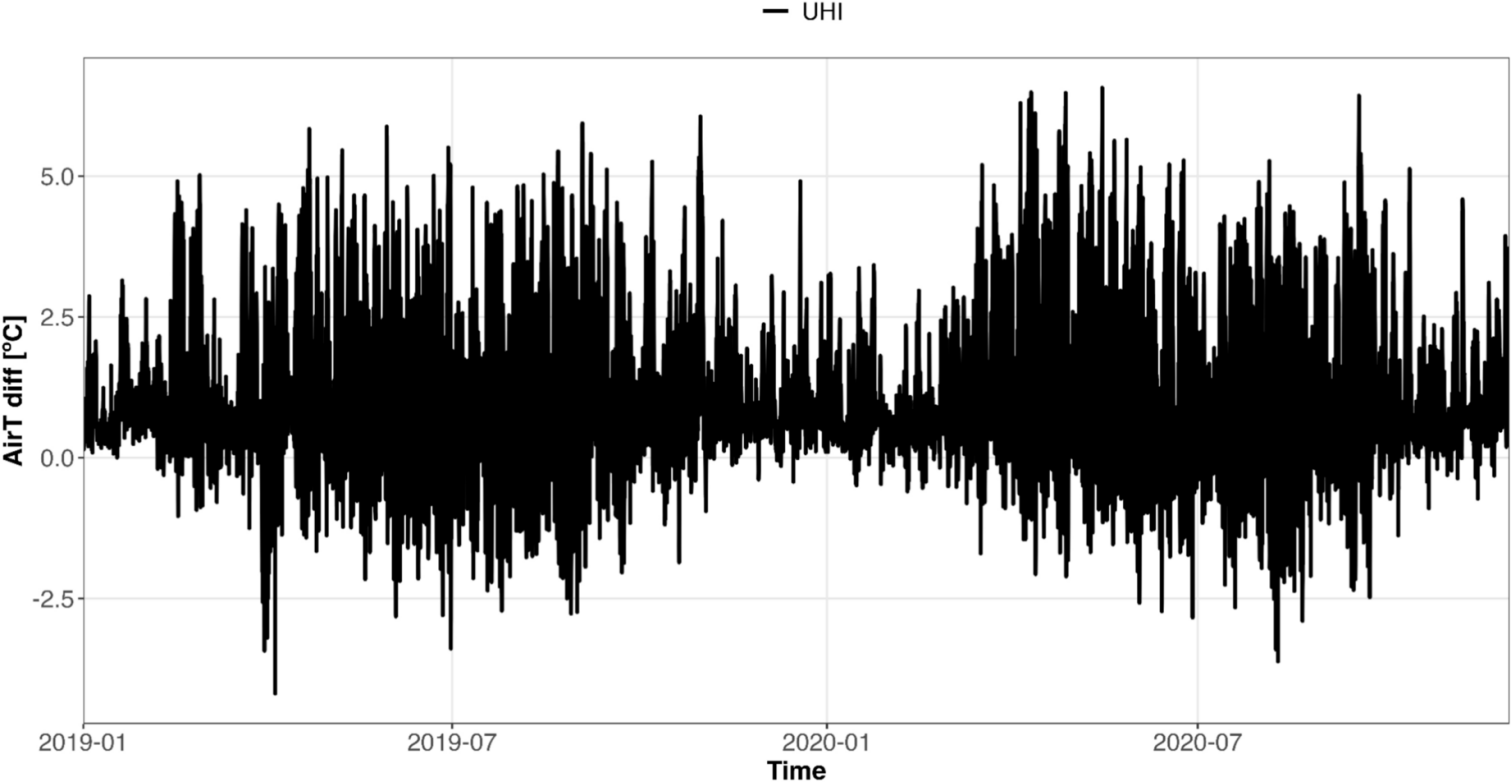
Time series of hourly UHI intensity in Berlin for 2019–2020 (local time) calculated using lcz_uhi_intensity( ) function from the LCZ4r package.

LCZ system provides a more conductive approach for UHI magnitude analysis compared to the commonly used urban–rural station differences^1^. LCZ4r therefore offers a functionality that calculates UHI intensity using temperature contrasts between distinct LCZ classes ($\underline{LCZ} x_{urban } - \underline{LCZ} y_{rural}$). By combining LCZ class with air temperature observations, this method reduces subjectivity and generates a reliable and comparable UHI dataset. For instance, Fig. 8 shows the hourly UHI intensity in Berlin from 2019 to 2020 and emphasizes how its magnitude fluctuates across seasons and daily cycles.

**Fig. 9:**
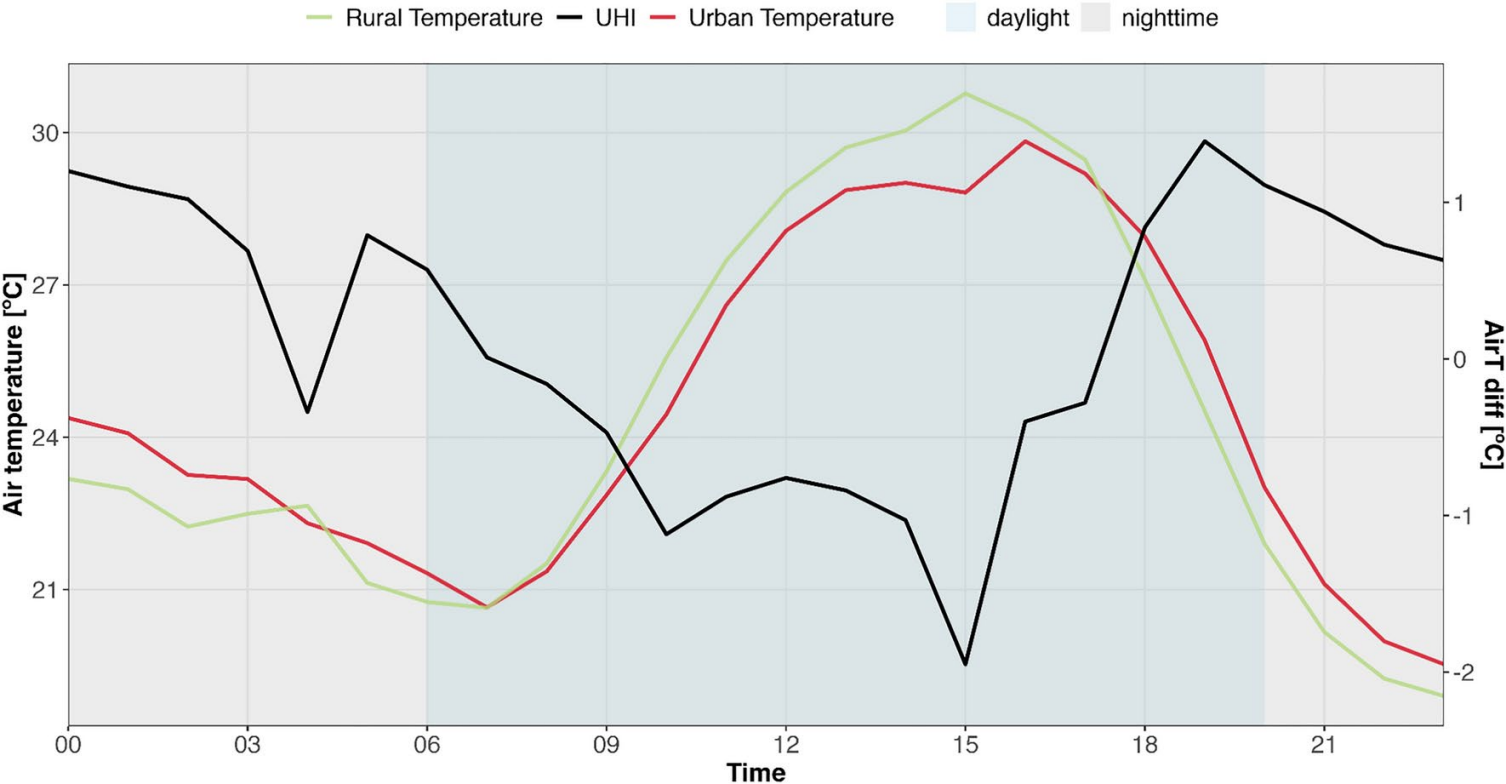
Daytime and nighttime variation of UHI intensity in Lisbon on July 4, 2017 (UTC) calculated using lcz_uhi_intensity(…, by = “daylight”) function from the LCZ4r package.

To analyze intra-day UHI variability, the lcz_uhi_intensity( ) function enables the assessment of UHI during specific time windows, such as daytime and nighttime. Figure 9 presents the UHI variation on a specific summer day in Lisbon. Note that the selected urban and rural temperatures are accompanied by their respective standard LCZ class colors. In this case, LCZ 2 (compact mid-rise) and LCZ 14 (low plants) were selected as references for UHI intensity calculations.

#### Interpolating air temperature with LCZ classification

The LCZ4r package offers two pivotal functions, lcz_interp_map and lcz_anomaly_map( ), designed to integrate LCZ framework into air temperature interpolation. These functions extend traditional kriging models by incorporating land-use and land-cover information specific to LCZ classes, in addition to spatial distances between measurement points. This novel approach enhances the spatial representation of thermal variability, resulting in a more realistic depiction of urban heat patterns that conventional methods often overlook.

**Fig. 10:**
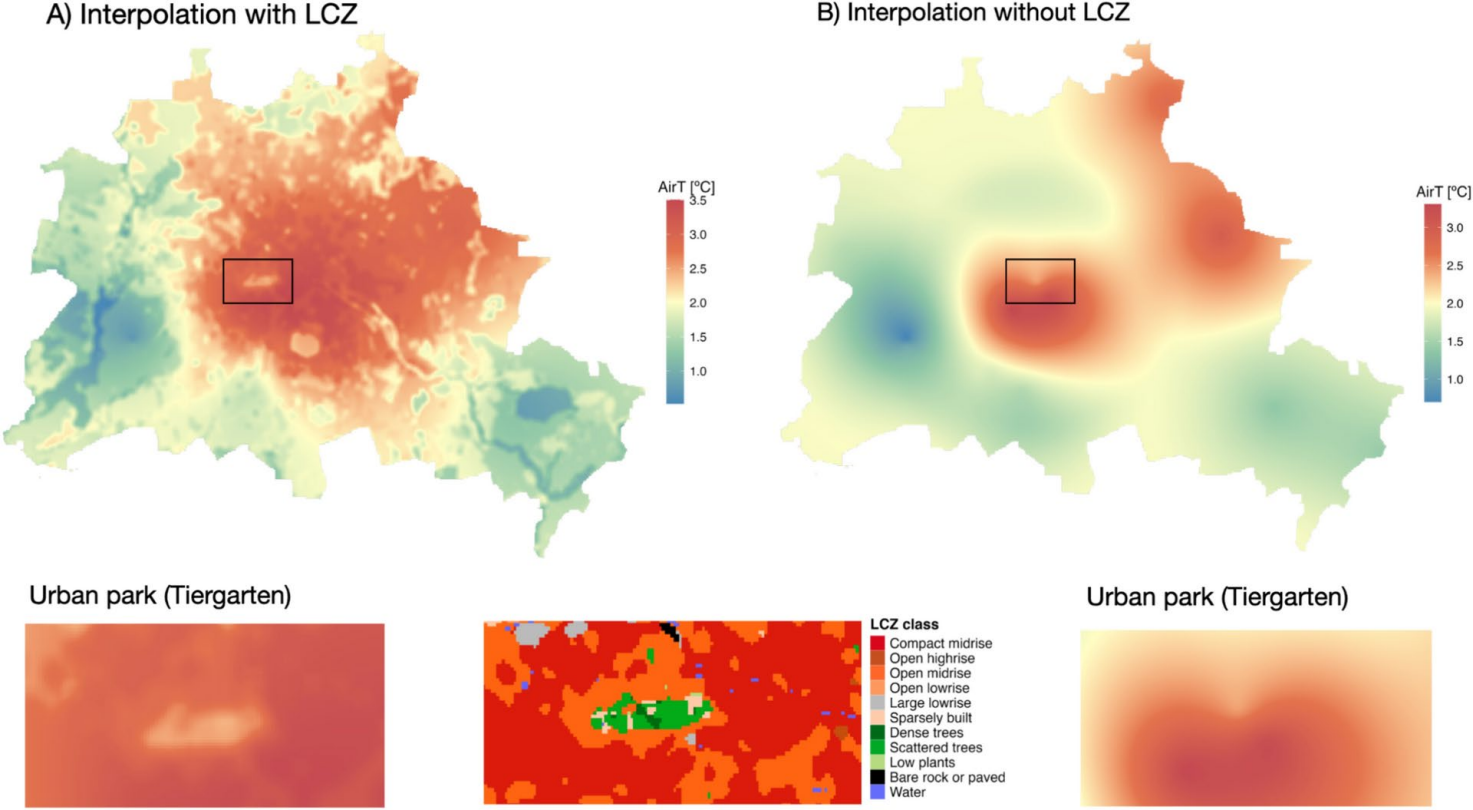
Air temperature interpolation at a 100 × 100 m resolution for Berlin on February 6, 2019, at 05:00 h (Local Time), with (A) LCZ classification and (B) without LCZ. The LCZ map was derived from the European LCZ product and generated using the lcz_get_map_euro( ) function. The interpolation was performed with the lcz_interp_map( ) function from the LCZ4r package using eight meteorological stations.

To demonstrate the benefits of using LCZ-based interpolation, we present two common scenarios found in most cities: Berlin, with a relatively modest density of urban stations (17 stations), and Curitiba, with limited measurement data (8 stations). Figure 10 illustrates the application of LCZ-based interpolation for Berlin during a winter hour on February 6, 2019, at 05:00 h. It reveals elevated air temperatures in densely built central areas, classified as LCZ 2 (compact midrise), LCZ 3 (compact low-rise), and LCZ 4 (open high-rise), as well as fine-scale thermal patterns influenced by urban features such as parks (e.g., Tiergarten) and water bodies. These patterns are clearly captured in LCZ-based interpolation (Panel A) but are largely absent in conventional interpolation without LCZ integration (Panel B).

**Fig. 11:**
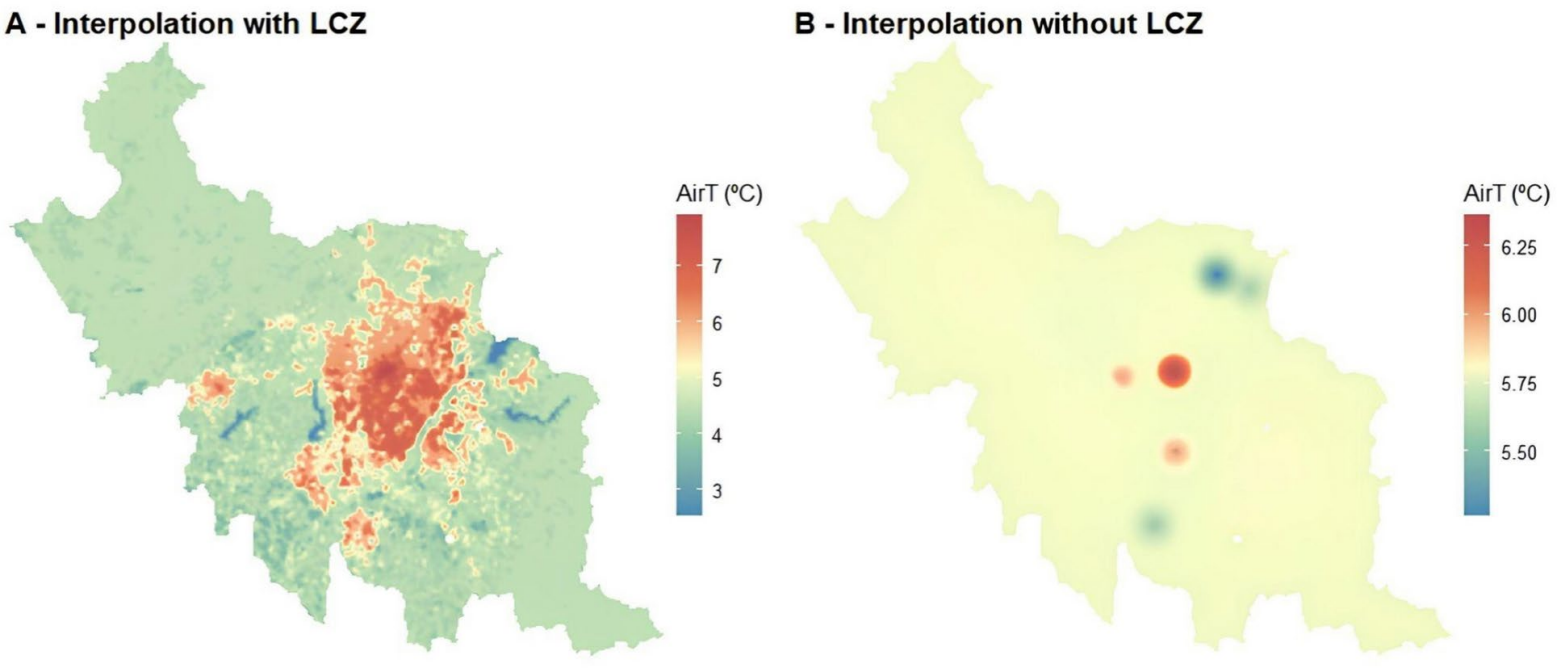
Daily air temperature interpolation at a 100 × 100 m resolution for Curitiba and metropolitan area on September 1, 2016 (Local Time), with (**A**) LCZ classification and (**B**) without LCZ. The LCZ map was derived from the Global LCZ product and generated using the lcz_get_map_euro( ) function. The interpolation was performed with the lcz_interp_map( ) function from the LCZ4r package using eight meteorological stations.

In the Curitiba and metropolitan area with a limited observational network (eight meteorological stations), LCZ-based interpolation demonstrated its capability to address data scarcity (Fig. 11). The interpolate map with LCZ produces smooth and realistic air temperature distributions, even in areas with no direct measurements (Panel A), as well as reduces the “bullseye effect”, a common problem in conventional interpolation methods caused by sparsely distributed stations (Panel B).

These examples highlight the potential of LCZ-based interpolation techniques to improve spatio-temporal representativeness of air temperature analysis across cities with varying station densities. A better spatial distribution of air temperature data helps researchers, urban planners, decision makers to take informed actions to combat the negative effects of the UHI.

#### Assessment of LCZ based-interpolation across observational scenarios

To evaluate the performance of the LCZ-based interpolation approach, we designed two scenarios reflecting real-world extremes in data availability: (1) an ultra-high-density observational scenario, which examines the method’s performance under ideal, data-rich conditions, and (2) a low-density observational scenario, designed to test the method’s generalization in data-limited situations, a common reality in many global regions. Subsequently, we presented a comparative analysis of the two scenarios, highlighting the strengths and limitations of the approach.

1. Ultra-high-density observational scenario

To test LCZ-based interpolation under data-rich conditions, we used daily air temperature data from 2, citizen weather stations (CWS) across Berlin 2018 (Figure SI.1 in Supplementary Information). These data, widely used in urban climate studies^36,45,46^, were processed using the statistically-based quality control method developed by Napoly et al.^47^, to identify and remove suspicious raw air temperature readings with the CrowdQC + package in R^48^, ensuring their suitability for assessing LCZ-based interpolation accuracy.

To correctly assign a pixel’s LCZ value and select the appropriate air temperature signal for each LCZ class, especially in areas with multiple CWS stations, we employed a two-step LCZ assessment method^49^ in the lcz_interp_eval( ) function. First, this method begins by filtering out stations based on their surrounding pixel environment; specifically, it analyzes a 5 × 5 pixel kernel (approximately 250 m around each station) and retains only those stations where at least 80% of the pixels within this kernel belong to the same LCZ class as the center pixel assigned to the respective station. Next, the LCZ value is assigned to the pixel based on the filtered stations, ensuring that the air temperature data used for interpolation represents local climate characteristics^49^.

**Fig. 12:**
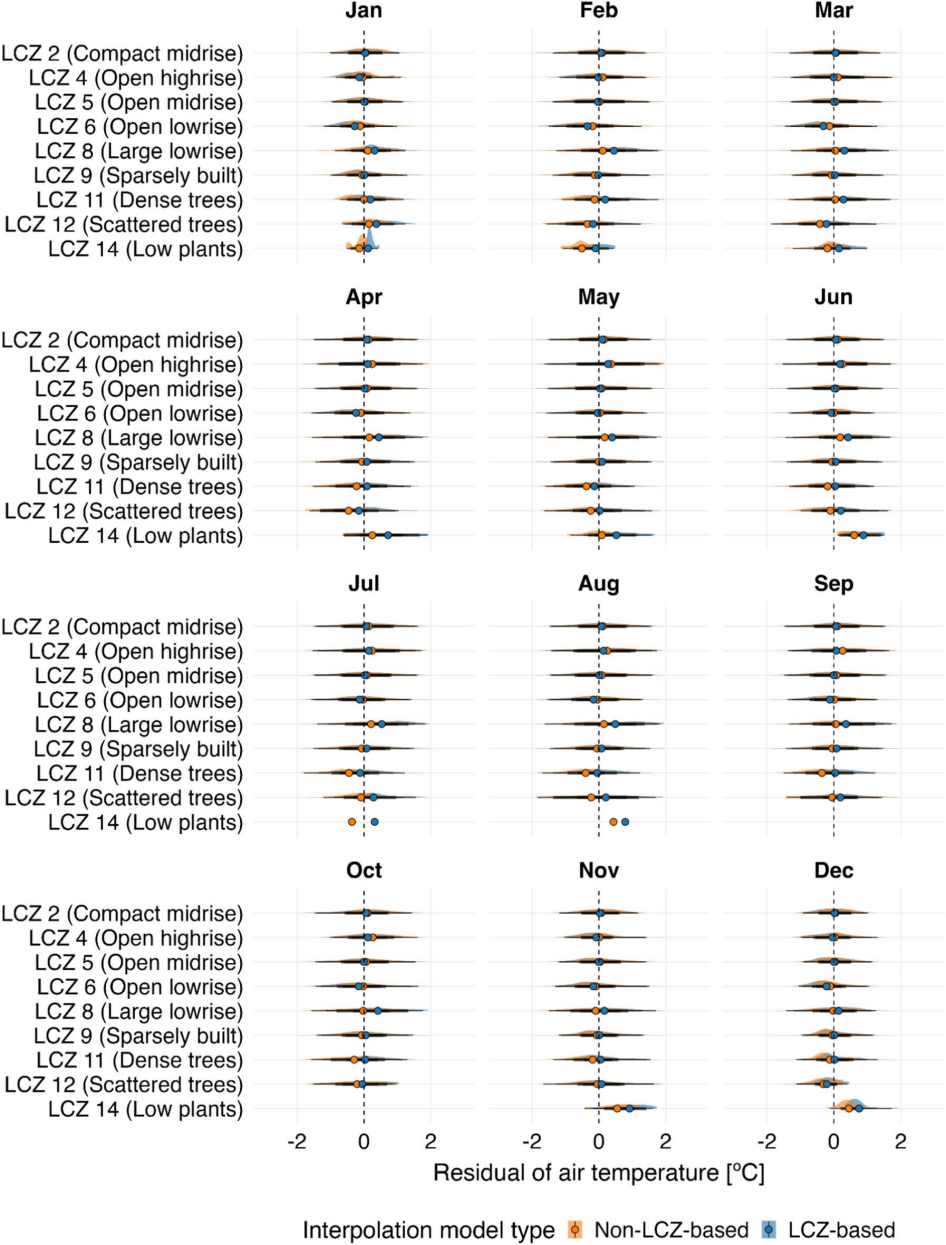
Daily distribution of air temperature residuals (°C) by month 2018 in Berlin (UTC + 1), comparing LCZ-based interpolation with conventional interpolation (Non-LCZ-based) for the ultra-high density observational scenario. Residuals represent the daily differences between observed and predicted air temperature values for both methods, with outliers excluded (IQR method). The residuals were generated using LOOCV and aggregated by LCZ class. Interpolated maps, created at a 500-m resolution, were produced using the LCZ Generator product via the lcz_get_map_generator( ) and lcz_interp_eval( ) functions from the LCZ4r package. Colored dots on the plot indicate the mean residuals.

Performance was assessed using Leave-one-out cross-validation (LOOCV), where each data point was sequentially removed, predicted using the remaining dataset, and compared to its observed value. The results, stratified by LCZ, are presented in Table 3 and Fig. 12.

**Table 3:** Statistical comparison of LCZ-based (LCZ + kriging) and conventional kriging only interpolation methods across selected LCZ classes under an ultra-high-density observational scenario (CWS data)

LCZ class	Number of observations	Interpolation method					
		LCZ-based			Conventional		
		RMSE (°C)	MAE (°C)	sMAPE (%)	RMSE (°C)	MAE (°C)	sMAPE (%)
2 (Compact midrise)	95,907	0.69	0.55	10.1	0.70	0.55	10.2
4 (Open high-rise)	3226	0.74	0.59	11.9	0.77	0.61	11.8
5 (Open midrise)	117,786	0.70	0.56	12.1	0.70	0.56	11.9
6 (Open low-rise)	14,754	0.68	0.54	14.6	0.65	0.51	13.8
8 (Large low-rise)	3086	0.84	0.68	13.0	0.75	0.61	11.7
9 (Sparsely built)	103,024	0.66	0.52	12.1	0.66	0.52	12.2
11 (Dense trees)	2214	0.64	0.50	12.5	0.70	0.56	13.2
12 (Scattered trees)	612	0.69	0.54	17.7	0.75	0.59	18.1
14 (Low plants)	109	0.78	0.62	25.2	0.59	0.47	21.3

Daily values for 2018 were aggregated by LCZ class. Key metrics include root mean square error (RMSE), mean absolute error (MAE), and symmetric mean absolute percent error (sMAPE). Outliers were excluded using the interquartile range (IQR) method, removing values beyond 1.5 times the IQR.

The LCZ-based method demonstrated superior performance in vegetation-dominated zones, outperforming conventional kriging in LCZ 11 by reducing RMSE by 8.6% (0.64 °C vs. 0.70 °C) and MAE by 10.7% (0.50 °C vs. 0.56 °C). In built-up areas (e.g., LCZ 2, 5, and 9), both methods exhibited comparable performance, indicating that LCZ integration offers limited additional benefits in these classes. However, the LCZ-based method faced challenges in morphologically heterogeneous or data-sparse zones, where conventional kriging achieved a 30.7% lower RMSE in LCZ 14 (0.59 °C vs. 0.78 °C) and an 11.9% improvement in LCZ 8.

Seasonally, the LCZ-based method consistently produced smaller mean residuals (centered near 0 °C) in LCZ 11 across all months, underscoring its ability to spatially represent the cooling effects of dense vegetation on local air temperatures (Fig. 12). In contrast, LCZ 8 and LCZ 14 exhibited larger residuals, particularly during the summer months (June–August), highlighting the persistent challenges of interpolating air temperatures in mixed and data-sparse zones.

These discrepancies can be attributed to unresolved air temperature signals from non-standard sensor placements near heat-retaining surfaces (e.g., rooftops, parking lots) in LCZ 8, as well as insufficient station coverage in LCZ 14 (n = 109), which hinders the accurate representation of air temperature patterns characteristic of these zones. In such cases, conventional kriging outperforms LCZ-based interpolation due to its reliance on spatial smoothing, which effectively averages out localized thermal variations. In contrast, the LCZ-based approach struggles to resolve mixed thermal patterns when station distribution does not align adequately with the distinct morphological features of the LCZs.

2. Low-density observational scenario

To further assess the LCZ-based method’s performance, we replicated the analysis under a low-density observational scenario using daily air temperatures from 23 meteorological stations in Berlin 2019 (Table 2) and the LOOCV method. Unlike the high-density scenario, we selected the simple LCZ pixel value assessment method, which assigns the LCZ class based on the raster cell in which a station point is located, as this approach is recommended for data-sparse contexts.

The LCZ-based interpolation showed marked improvements in some LCZ classes but limitations in others (Table 4). For instance, in LCZ 2, the LCZ-based approach outperformed the conventional method, reducing RMSE by 20.8% (0.52 °C vs. 0.65 °C). In vegetation-dominated LCZs (e.g., LCZ 11 and 12), the LCZ-based method further demonstrated its potential even under data-scarce conditions. However, in LCZ 14 and LCZ 16, conventional kriging outperformed LCZ-based interpolation, reducing RMSE by 29.5% (0.40 °C vs. 0.56 °C) and 34.2% (0.40 °C vs. 0.61 °C), respectively, likely due to the combined effects of sparse station density and complex thermal patterns.

**Table 4:** Statistical comparison of LCZ-based (LCZ + kriging) and conventional with only kriging interpolation methods under a low-density observational scenario across selected LCZ classes.

LCZ class	Number of observations	Interpolation method					
		LCZ-based			Conventional		
		RMSE (°C)	MAE (°C)	sMAPE (%)	RMSE (°C)	MAE (°C)	sMAPE (%)
2 (Compact midrise)	330	0.52	0.43	7.6	0.65	0.59	10.3
5 (Open midrise)	1790	0.53	0.42	7.9	0.48	0.38	7.4
9 (Sparsely built)	365	0.37	0.29	4.9	0.36	0.28	4.6
11 (Dense trees)	1403	0.46	0.37	8.1	0.48	0.39	8.7
12 (Scattered trees)	1063	0.39	0.30	5.6	0.43	0.34	6.6
14 (Low plants)	712	0.56	0.47	9.6	0.39	0.31	6.0
16 (Bare soil or sand)	348	0.61	0.51	11.5	0.40	0.31	6.9

Daily values for 2019 were aggregated by LCZ class.

**Fig. 13:**
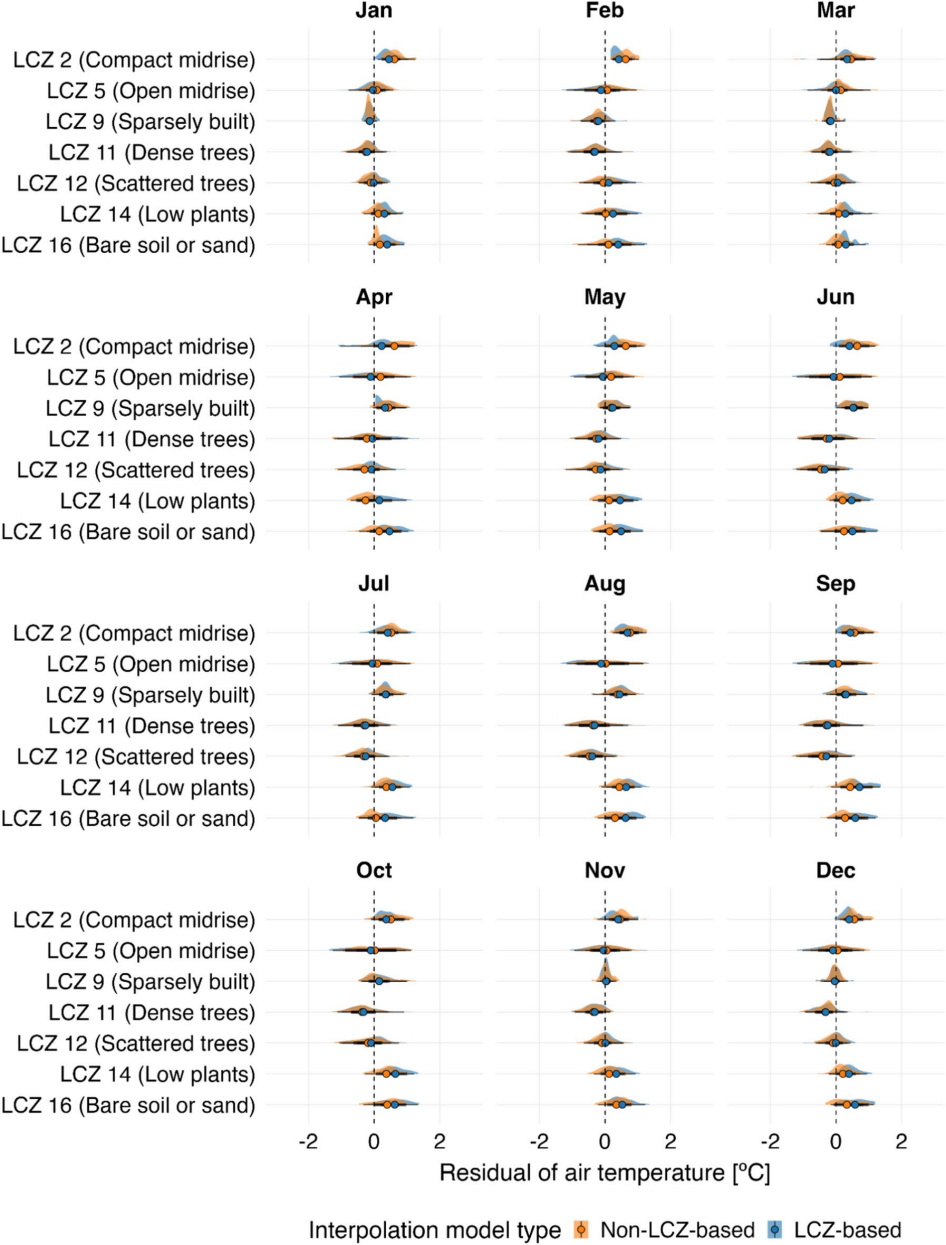
Daily distribution of air temperature residuals (°C) by month 2019 in Berlin (UTC + 1), comparing LCZ-based interpolation with conventional with only kriging (Non-LCZ-based) for the low-density observational scenario. Residuals represent the daily differences between observed and predicted air temperature values for both methods, with outliers excluded (IQR method). The residuals were generated using LOOCV and aggregated by LCZ class. Interpolated maps, created at a 500-m resolution, were produced using the LCZ Generator product via the lcz_get_map_generator( ) and lcz_interp_eval( ) functions from the LCZ4r package. Colored dots on the plot indicate the mean residuals.

When analyzing seasonal residuals (Fig. 13), we observed that the LCZ-based approach generally exhibited values more tightly clustered around zero, with lower dispersion across all months. This pattern was particularly evident in densely built urban zones (e.g., LCZ 2), likely due to more accurate air temperature representation from properly sited stations.

3. Comparative analysis of both scenarios

Overall, our results demonstrate that LCZ-based interpolation can enhance temperature representation in vegetation-dominated areas (e.g., LCZ 11) and densely built areas with representative stations (e.g., LCZ 2), even under low-density scenarios, which is a critical advantage for UHI analysis and green infrastructure planning. However, its efficacy declines in data-sparse or morphologically complex areas (e.g., LCZ 8, 14, 16). Future studies could explore hybrid approaches that integrate LCZ classification with advanced machine learning (ML) techniques to improve generalization in heterogeneous urban environments.

To place our findings in a broader context, the performance metrics of LCZ-based interpolation metrics derived from both ultra-high and low density scenarios are consistent with those reported in air temperature interpolation studies^50,51^ (see Table SI.1), where RMSE values range from 1.06 to 2.99 °C and MAE from 0.7 to 2.29 °C. However, direct comparisons should be made with caution due to differences in evaluation procedures, instrumentation, data collection periods, sensor placement, and prevailing climatic conditions, all of which can influence interpolation outcomes.

While the findings reveal the potential of LCZ-based interpolation, two caveats should be noted. First, it is important to acknowledge that LCZ based-interpolation may not fully account for localized temperature variation influenced by natural and anthropogenic factors beyond the scope of LCZ classification^52^. Users should thus assume the LCZ system as one of several contributors to apparent air temperature differences. To minimize this inherent limitation, it is recommended to conduct preliminary data exploration with functions such as lcz_ts( ) and lcz_anomaly( ), which provide an initial temporal variation of temperatures across LCZ classes, or integrating domain-specific knowledge about the study area.

Second, the performance of LCZ-based interpolation is influenced by multiple factors, including the quality data, resolution of LCZ maps, urban morphology, and meteorological conditions. To address these issues, we emphasize the importance of applying the LCZ4r tool in diverse climate contexts. In this regard, user contributions are necessary for validating the tool’s performance and driving its ongoing improvement.

## Application of LCZ4r tool

### Urban planning and sustainable development actions

At the urban scale, understanding timing, location, and nature of LCZ-related thermal patterns is critical for informed climate actions. Accurate and high-resolution climate maps are indispensable tools for researchers, urban planners, and policymakers to evaluate and implement strategies aimed at mitigating UHI effects^53^. The LCZ4r offers a toolkit tailored to support these needs, combining general and local functions with practical applications for urban sustainability studies^54^. For instance, lcz_uhi_intensity( ) and lcz_interp_map( ) allow for quantification and spatial–temporal representation of UHI configurations and intensities, aiding in the identification of thermal hotspots for targeted interventions. Historical and future LCZ dynamics can be explored using lcz_cal_area( ) and lcz_ts( ), enabling users to track urbanization trends and project the impacts of land-use changes. Moreover, lcz_interp_map( ) supports the assessment of air temperature and air pollution patterns, even in complex urban environments where microclimatic variations are pronounced. Functions such as lcz_anomaly( ) and lcz_anomaly_map( ) extend the analysis to thermal comfort and health-related metrics. And, the integration of lcz_get_parameters( ) with lcz_uhi_intensity( ) provides respective UCP and UHI information for estimating energy consumption and carbon emissions, essential for designing low-carbon cities.

### Climate modeling and machine learning

The high-resolution, accurate, and informed UHI and UCP maps generated by the LCZ4r tool can be seamlessly integrated into climate modeling frameworks^55^. These outputs serve as inputs for developing predictive models of urban thermal environments^7^ and for linking LCZ data with biophysical models to estimate heat, CO_2_, and water vapor fluxes in urban settings^56^. Besides, the data derived from the LCZ4r tool, such as interpolated hourly air temperatures or specific UCP grids, can be incorporated into ML workflows that model urban environment dynamics. The application of ML in climate modeling and weather prediction is increasingly recognized for its potential to leverage large datasets^57^. With the high-resolution UHI grids embedded in the LCZ4r tool, researchers can benefit from more accessible urban climate-related features, boosting the performance of ML parameterization models^58^.

## Version limitations

The LCZ4r package in its 0.1.0 version has a few limitations that should be considered. While LCZ4r has shown consistent and reliable performance in generating LCZ maps and outcomes at the municipal and state scales, the LCZ map extraction procedure has led to a massive processing and memory allocation issues at regional and national scales, causing instability in general and local functions.

Users should make sure when using the lcz_get_* functions with OSM identification labels (tags, ids, names), as discrepancies can arise when comparing official zoning, boundaries, and borders with community-generated OSM data. Furthermore, OSM data may have limited spatial coverage in certain areas, making it impossible to obtain LCZ map data. In cases where LCZ4r does not work well, particularly in oceanic and island regions, it is recommended that users utilize their own LCZ map raster and files in shapefile format through the ROI argument of the lcz_get_* functions.

## Future developments

The LCZ4r package is an open-source and collaborative tool. All its functions are hosted on the GitHub platform, where users can become contributors, allowing them to access the R codes and make changes to specific functions of the package (https://github.com/ByMaxAnjos/LCZ4r/). We highly value user feedback and encourage users (and contributors) to share new ideas and report any issues while using the LCZ4r. This feedback is essential in helping us improve the tool in future versions.

There are at least three plans for the future development of LCZ4r. First, while the current version of LCZ4r focuses on the UHI urban canopy layer, our next version aims to broaden the scope of UHI analysis. Specifically, we plan to develop a function, lcz_uhi_surface( ), that generates Land Surface Temperature (LST) for any land area worldwide. This new functionality will enable users to obtain LST data for a wide range of locations, facilitating comprehensive UHI analysis beyond the height-screen air temperatures.

The second plan is to enhance the accessibility and usability of the LCZ classification by developing a multilingual version and integrating LCZ4r software into a user-friendly Geographic Information System (GIS) interface (https://bymaxanjos.github.io/LCZ4r/articles/Introd_QGIS_LCZ4r.html). By adding the script of LCZ4r functions into ARCGIS and QGIS, we can enhance the use of LCZ data via the Graphical User Interface (GUI) environment. This multilingual integration would be beneficial for users from diverse linguistic and cultural backgrounds who are not familiar with programming through a command-line interface.

Third, we plan to also translate the LCZ4r package to other programming languages, such as Python and Matlab. By doing so, we aim to increase the accessibility of LCZ data and modeling of the UHI to a broader range of coding users. This step, including the LCZ4r-GIS bridge, will allow millions of users to utilize LCZ4r and contribute to LCZ and UHI analysis across different programming platforms and applications.

## Supplementary Information

Supplementary Information.

## Data Availability

All the codes used in this study have been uploaded on the same public GitHub repository (https://github.com/ByMaxAnjos/LCZ4r).
