# Supplementary material for: LCZ4r package R for local climate zones and urban heat islands
Source: Sci Rep. 2025 Mar 5;15:7710. doi: 10.1038/s41598-025-92000-0 (PMC11882999; doi:10.1038/s41598-025-92000-0)
Supplement: Supplementary file 1 — Supplementary Information. [file 41598_2025_92000_MOESM1_ESM.docx]

Supplementary Information for

**LCZ4r package R for Local Climate Zones and Urban Heat Islands**

*Max Anjos^a,b^, Dayvid Medeiros^a^, Francisco Castelhano^a^, Fred Meier^b^, Tiago Silva^c,d^ , Ezequiel Correia^c,d^, and António Lopes^c,d^

^a^Department of Geography, Federal University of Rio Grande do Norte, Brazil.

^b^Chair of Climatology, Institute of Ecology, Technische Universität Berlin, Rothenburgstraße 12, 12165 Berlin, Germany.

^c^University of Lisbon, Institute of Geography and Spatial Planning (IGOT), Centre of Geographical Studies (CEG), Lisbon, Portugal.

^d^Associate Laboratory Terra, Portugal

Corresponding author

*Max Anjos, [maxanjos@campus.ul.pt](mailto:maxanjos@campus.ul.pt)

**Figure SI.1**: (A) Berlin LCZ map used in the interpolation and generated with LCZ Generator product[^1^](https://www.zotero.org/google-docs/?aeIEMc) via the lcz_get_map_generator( ) from LCZ4r R package. **(B)** Geographical distribution of filtered 2,474 citizen weather stations in Berlin. Base map tiles by OpenStreetMap (© OpenStreetMap contributors, www.openstreetmap.org/copyright), rendered using the Leaflet library.

**A)**

**
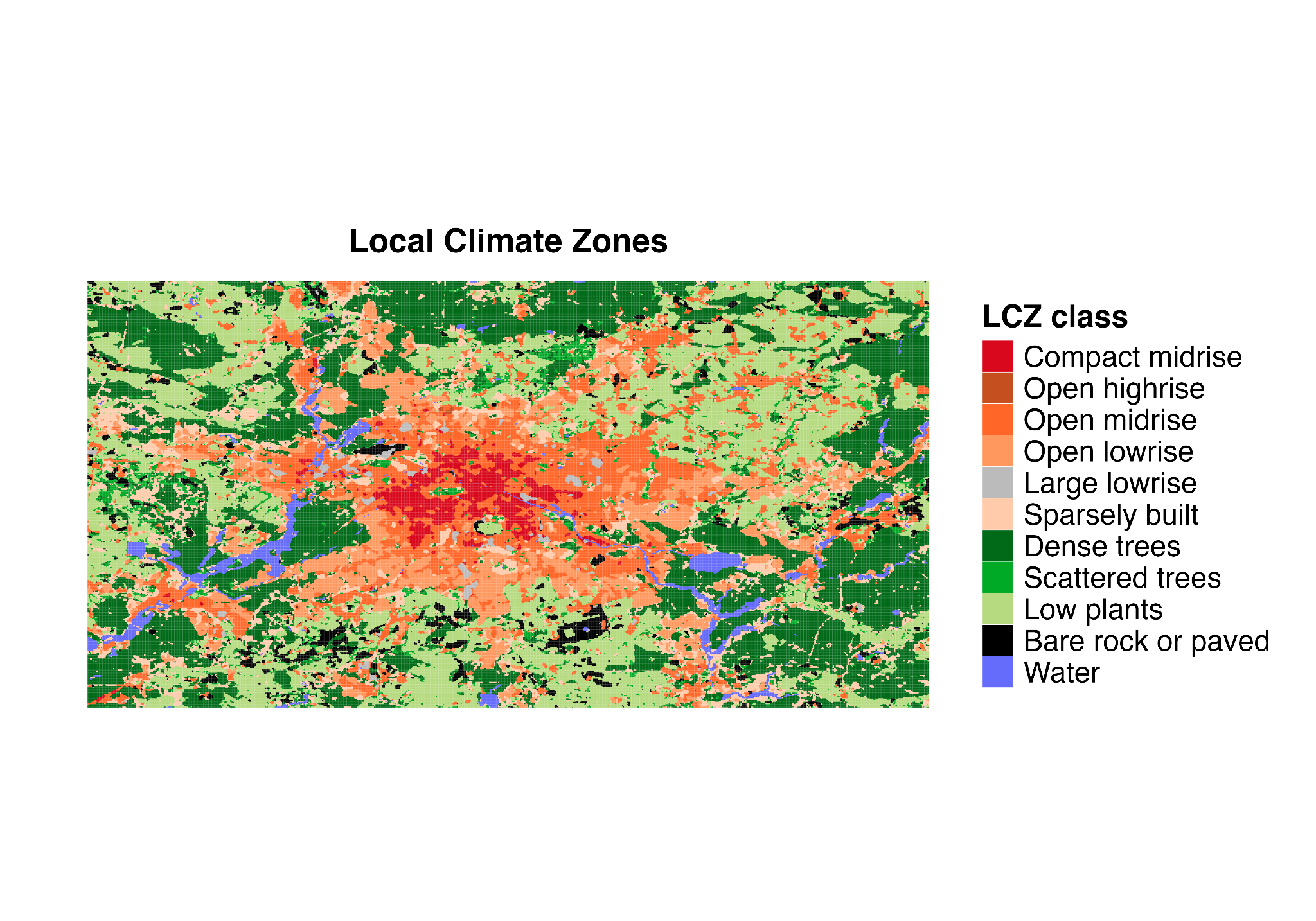
**

**B)**

**
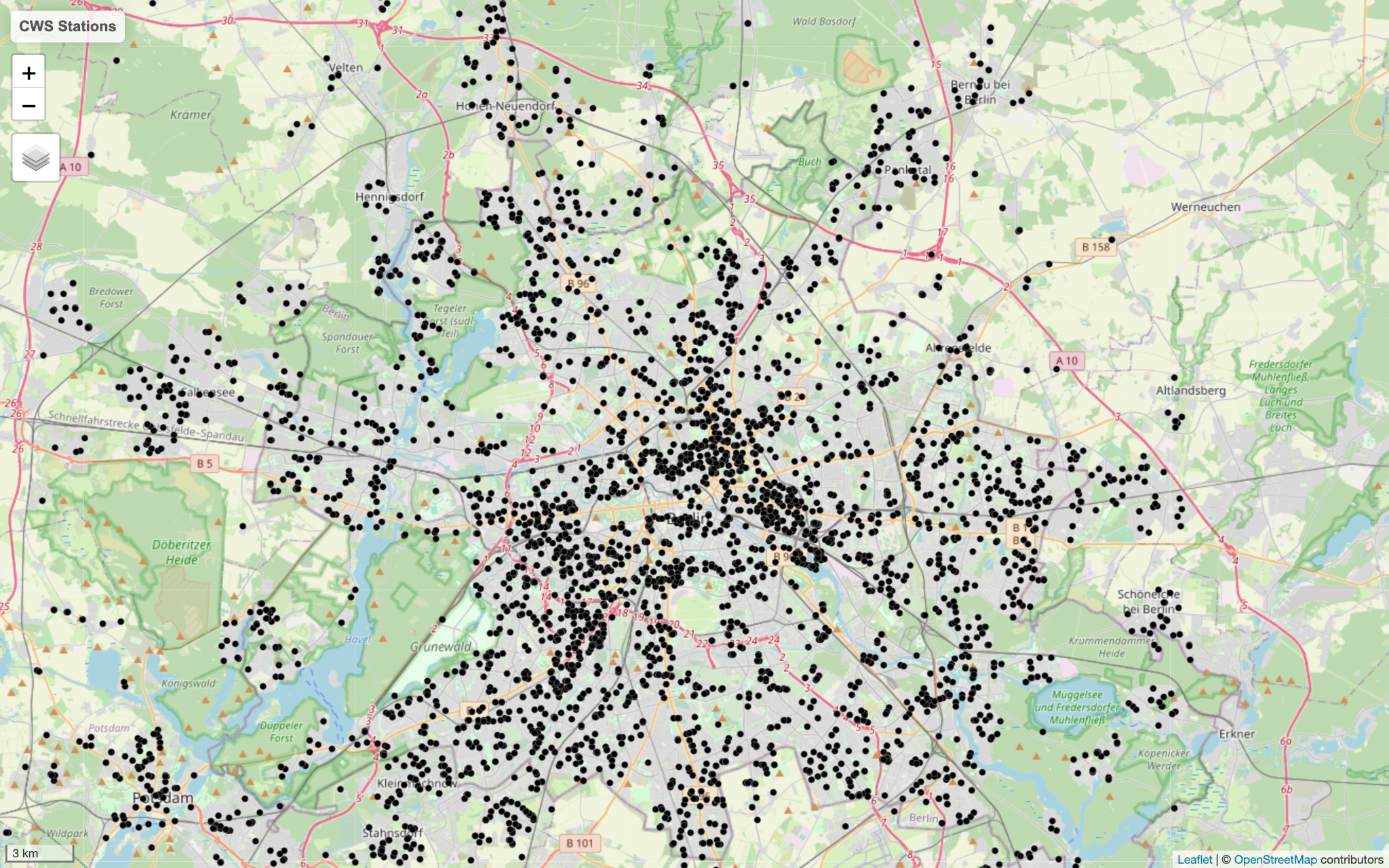
**

**Table SI.1**: Summarize studies and various interpolation methods applied to air temperature data, with their respective data, spatiotemporal resolution, and accuracies measured by RMSE and MAE. Note that RMSE and MAE values from LCZ-based interpolation were calculated to 1 km spatial resolution.

| **Interpolation Method** | **Data** | **Spatial Resolution** | **Temporal Resolution** | **RMSE**  ( °C) | **MAE**  ( °C) | **Reference** |
| --- | --- | --- | --- | --- | --- | --- |
| LCZ-Ordinary kriging | Netatmo crowdsourced weather station data in Berlin, Germany | ~1 km | Daily  (Jan - Dec 2018) | 0.91 | 0.67 | This study |
| Ordinary Kriging | Netatmo crowdsourced weather station data in Warsaw, Poland. | ~1 km | Daily (Jun–Sep) | 1.06 | -- | Hassani et al.,[^2^](https://www.zotero.org/google-docs/?adHpWS) |
| Machine Learning (ML) Model | Same as above. | ~1 km | Daily (Jun–Sep) | 1.23 | – | Hassani et al.,[^2^](https://www.zotero.org/google-docs/?dZuxqz) |
| Weather Research and Forecasting (WRF) Model | Same as above. | ~1 km | Daily (Jun–Sept) | 1.7 | – | Hassani et al.,[^2^](https://www.zotero.org/google-docs/?2jSXKY) |
| Deep Belief Network (DBN) | Various data sources across China. | 0.01° (~1.1 km) | Daily | 1.996 | 1.539 | Shen et al.,  [^3^](https://www.zotero.org/google-docs/?nR6vIR) |

**References**

[1. Demuzere, M., Kittner, J. & Bechtel, B. LCZ Generator: A Web Application to Create Local Climate Zone Maps. *Front. Environ. Sci.* **9**, 637455 (2021).](https://www.zotero.org/google-docs/?FObXpA)

[2. Hassani, A., Santos, G. S., Schneider, P. & Castell, N. Interpolation, Satellite-Based Machine Learning, or Meteorological Simulation? A Comparison Analysis for Spatio-temporal Mapping of Mesoscale Urban Air Temperature. *Environ. Model. Assess.* **29**, 291–306 (2024).](https://www.zotero.org/google-docs/?FObXpA)

[3. Shen, H. *et al.* Deep learning-based air temperature mapping by fusing remote sensing, station, simulation and socioeconomic data. *Remote Sens. Environ.* **240**, 111692 (2020).](https://www.zotero.org/google-docs/?FObXpA)
